# First case of human infection with *Plasmodium knowlesi* in Laos

**DOI:** 10.1371/journal.pntd.0006244

**Published:** 2018-03-22

**Authors:** Moritoshi Iwagami, Masami Nakatsu, Phonepadith Khattignavong, Pheovaly Soundala, Lavy Lorphachan, Sengdeuane Keomalaphet, Phonepadith Xangsayalath, Satoru Kawai, Bouasy Hongvanthong, Paul T. Brey, Shigeyuki Kano

**Affiliations:** 1 Department of Tropical Medicine and Malaria, Research Institute, National Center for Global Health and Medicine, Tokyo, Japan; 2 SATREPS project (JICA/AMED) for Parasitic Diseases, Vientiane, Lao PDR; 3 Parasitology Laboratory, Institut Pasteur du Lao PDR, Ministry of Health, Vientiane, Lao PDR; 4 National Institute of Public Health, Ministry of Health, Vientiane, Lao PDR; 5 Department of Tropical Medicine and Parasitology, Dokkyo Medical University, Tochigi, Japan; 6 Center of Malariology, Parasitology and Entomology, Ministry of Health, Vientiane, Lao PDR; 7 Institut Pasteur du Lao PDR, Ministry of Health, Vientiane, Lao PDR; George Washington University School of Medicine and Health Sciences, UNITED STATES

## Presentation of case

On July 17, 2016, a 12-year-old Laotian boy living in Bengvilay village, Sanamxay district, Attapeu province (adjacent to Cambodia) in Laos visited Bengvilay Health Center. He manifested malaria-like symptoms and signs such as high fever (40 ^o^C), shivering, and nausea. The patient did not have a history of traveling outside of Attapeu province, Laos. The fever started 3 days before he visited the health center. He was diagnosed as having been infected with *Plasmodium vivax* malaria by a Rapid Diagnostic Test (RDT) kit (Malaria Ag *Pf*/*Pv*, Standard Diagnostics, Republic of Korea) at the health center, and a 3-day course of artemether/lumefantrine was prescribed. The patient recovered after the treatment. Prior to this episode, the patient had been recorded as having 4 previous malaria episodes that were diagnosed by the RDT kit, but detailed information, such as *Plasmodium* species and symptoms, was not recorded. The family used insecticide-treated bednets inside their home. The family was employed in agriculturally related jobs and occasionally went to the forest to collect foods and other necessities. The patient sometimes accompanied his family to the forest. A finger-prick blood sample was collected on a filter paper (Whatman FTA Classic Cards, GE Healthcare Life Science, United Kingdom) prior to the treatment, and we implemented a molecular approach to confirm the *Plasmodium* species by PCR and DNA sequencing.

We collected 2,698 finger-prick blood samples from malaria patients (positive by microscopy or RDT), including the 12-year-old Laotian boy, on the filter papers from 155 public health care facilities in the 5 southern provinces in Laos from October 2015 through October 2016 for detecting human infection with *P*. *knowlesi*. We examined all the blood samples by PCR.

## Approach

### Ethics statement

The research proposal was reviewed and approved by the National Ethics Committee for Health Research, Lao Ministry of Health (No. 049 NIOPH/NECHR), in 2014. Written informed consent was obtained from the patient’s father prior to the interview and the collection of blood for DNA analysis of malaria.

### DNA analysis

DNA was extracted from the dried blood spot on the filter paper with a Maxwell RSC Instrument (Promega, United States of America) in accordance with the manufacturer’s instructions with minor modification (S1 Table).

The *Plasmodium* species present in the patient’s blood were identified by real-time PCR using species-specific primers for 5 different *Plasmodium* species (S1 Table). Based on the real-time PCR, it was suspected that the DNA sample contained *P*. *knowlesi*. To confirm the *P*. *knowlesi* infection, DNA sequencing was conducted. First, 2 gene regions (partial *cytochrome b gene* [*cytb*] of the mitochondrial genome and *merozoite surface protein-1 gene* [*msp1*] of the nuclear genome) of *P*. *knowlesi* were amplified by nested PCR [1] (S1 Table). Second, the PCR amplicons were purified with Performa DTR Gel Filtration Cartridges (Edge Bio, United States of America) and sequenced by an ABI Genetic Analyzer model 3130XL (Life Technologies, Japan). Molecular phylogenetic trees were constructed based on polymorphic sites in the partial sequences of both gene regions using the neighbor-joining method in molecular evolutionary genetics analysis (MEGA) software version 7.0.21 [2]. In addition to sequences from the patient, those from *P*. *knowlesi* isolates from other countries and some other *Plasmodium* species that appear in GenBank were included in the tree.

**Fig 1:**
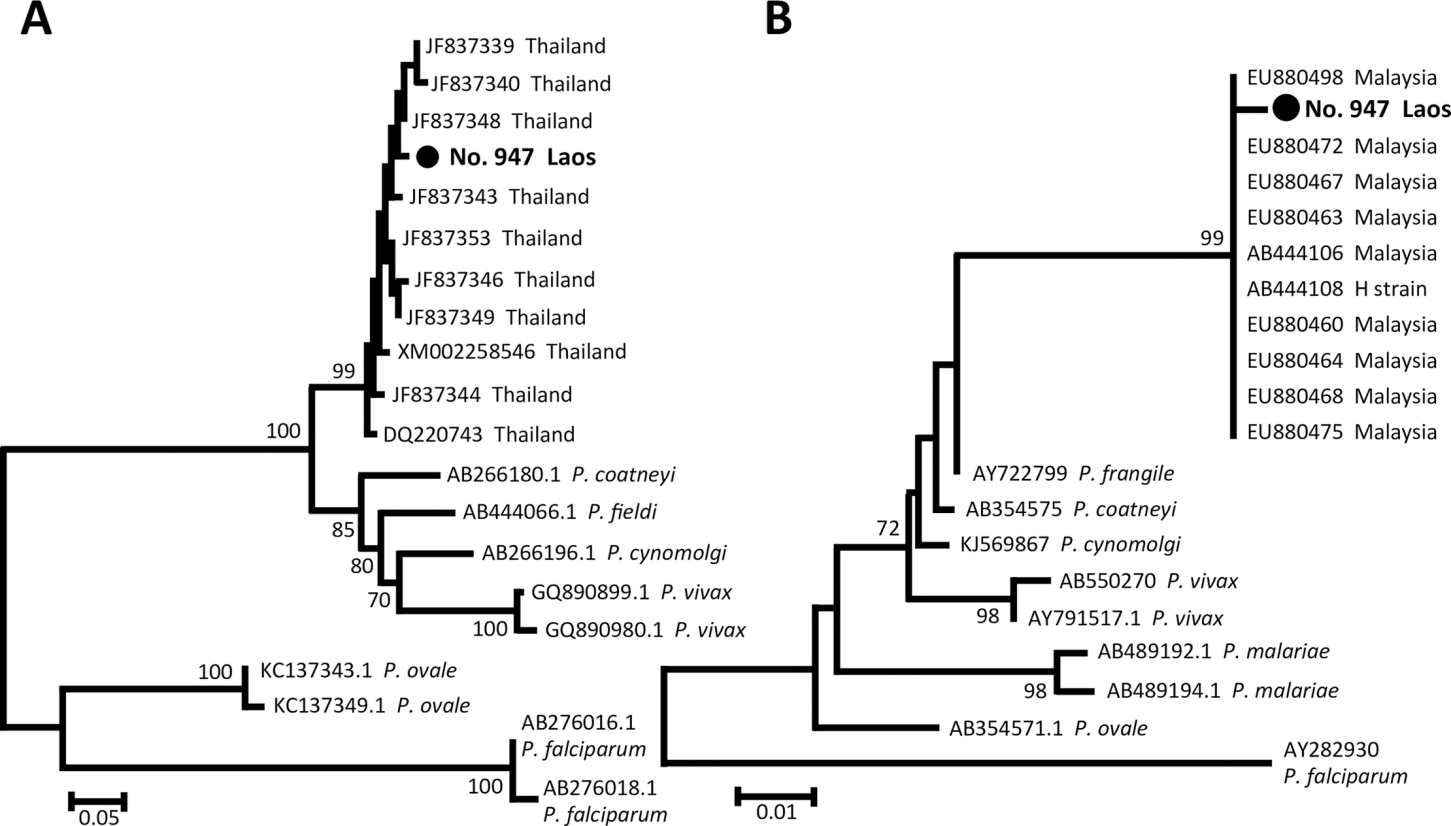
Phylogenetic analyses of a *Plasmodium knowlesi* isolate collected from a malaria patient in the south of the Lao PDR. **(A)** The phylogenetic tree of *P*. *knowlesi* and other malaria parasites inferred using partial *msp1* DNA sequences. **(B)** The phylogenetic tree of *P*. *knowlesi* and other malaria parasites inferred using partial *cytb* DNA sequences. The analysis was conducted using MEGA software version 7.0.21 [2] (http://www.megasoftware.net) by the neighbor-joining algorithm with Kimura’s 2-parameter model. The bootstrap values from 1,000 replicates are shown at node. Scale bars represent number of nucleotide substitutions per site. GenBank accession numbers and country of origin are shown in related sequences. “No. 947 Lao PDR” indicates the sample sequenced in this study. For the *msp1* tree, sequences of *P*. *fieldi*, *P*. *coatneyi*, *P*. *cynomolgi*, *P*. *vivax*, *P*. *malariae*, and *P*. *falciparum* were used as the out-group of the analysis. For the *cytb* tree, sequences of *P*. *frangile*, *P*. *coatneyi*, *P*. *cynomolgi*, *P*. *vivax*, *P*. *malariae*, and *P*. *falciparum* were used as the out-group of the analysis. The GenBank accession number and country of origin with no species name indicates the sequence of *P*. *knowlesi*. DNA sequences of the partial *cytb* and *msp1* were deposited in the DDBJ, for which accession numbers were LC327233 and LC327234, respectively. *cytb*, cytochrome b gene; DDBJ, DNA Data Bank of Japan; MEGA, molecular evolutionary genetics analysis; *msp1*, merozoite surface protein-1 gene.

In the phylogenetic analysis, the partial DNA sequences of the genes from the patient isolate clustered with the sequences of *P*. *knowlesi* isolates from Thailand or Malaysia (Fig 1). The *cytb* sequence of the Lao isolate (DNA Data Bank of Japan [DDBJ] accession no. LC327233) was 99% (221/223 bp) identical to that of a Malaysian isolate (GenBank accession no. EU880463), whereas the *msp1* sequence of the Lao isolate (DDBJ accession no. LC327234) was 99% (473/479 bp) identical to that of a Thai isolate (GenBank accession no. JF837343) (Fig 1).

## Case discussion

In this study, we identified the first documented case of a human infection with *P*. *knowlesi* in Laos, although it was a very rare case (1/2,698 cases). In this study, the predominant species were *P*. *falciparum* (42%), *P*. *vivax* (39%), and mixed infection with *P*. *falciparum* and *P*. *vivax* (4%).

The simian malaria parasite *P*. *knowlesi* is prevalent in macaque monkeys in Southeast Asian countries. Human infections with this parasite were thought to be extremely rare until multiple human infections were reported in 2004 from Malaysian Borneo [3]. Thereafter, several cases of human infections, including fatal cases, were reported in several Southeast Asian countries other than Laos [3–7]. Recently, however, a malaria study of long-tailed macaques in Southeast Asian countries demonstrated that 1 of 44 (2.3%) macaques originating from Laos was infected with *P*. *knowlesi*, but the exact geographic location where the infected macaque was captured in Laos was not given [8].

Presently, in Laos, the majority of malaria patients are adult males [9]. Higher numbers of malaria cases in men are usually associated with occupational activities, such as logging, mining, and dam or road construction in and through the forest [9]. In the present case, we speculate that the 12-year-old boy contracted *P*. *knowlesi* while going to the forest or somewhere near his village, which is surrounded by the forest and where wild monkeys had been observed previously (personal communication, father of the patient to the author). This case suggests that *P*. *knowlesi* is being transmitted in the forest near the village, but some malaria patients in the area may be contracting it without their knowledge, because no PCR analysis is available at the local health center. In addition, nowadays, ecotourism activities such as elephant riding, kayaking, trekking, caving, observing wild animals, and staying overnight in treehouses in the forest have become very popular among foreign travelers in Laos. These trends increase the number of foreign travelers who are in close contact with the forest and its fauna, including wild monkeys. Furthermore, a recent study reported that 63% (28/44) of long-tailed macaques in Laos were infected with *P*. *cynomolgi*, which can also be infectious to humans [8,10]. Therefore, an investigation of human infection with *P*. *cynomolgi* is also needed in Laos.

In this study, the patient was first diagnosed with *P*. *vivax* infection by the RDT kit. The positive *P*. *vivax* RDT result could have originated from a cross-reaction. In fact, previous studies demonstrated that false-positive results for *P*. *vivax* and *P*. *falciparum* have been observed for *P*. *knowlesi* mono-infection with certain RDTs [11,12]. The RDT sensitivity to *P*. *knowlesi* should be investigated further.

To better understand the epidemiology of simian malaria in Laos, further investigations, such as mass blood surveys among the villagers, entomological surveys to identify the species of *Anopheles*, and wild monkey surveys (using blood, urine, or feces), are needed.

**Key learning points**

- Given the limitations of the malaria RDT kit used in Laos, human infection with *Plasmodium knowlesi* is likely underdiagnosed in the country.
- As *P*. *knowlesi* infection has the potential to follow a severe (even fatal) course, careful treatment and follow-up are required even for patients diagnosed with vivax malaria based on an RDT kit.
- Travelers returning from Laos have a potential risk of infection with *P*. *knowlesi*, especially those who visited the forest or a village near the forest.

## Supporting information

S1 Table PCR primers, master mix, and condition of assay. Table footnotes: DNA extraction: DNA was extracted from the dried blood spots on the filter papers with a Maxwell RSC Instrument (Promega, United States of America) in accordance with the manufacturer’s instructions with minor modification. Three punched-out circles of 3.175-mm (1/8-inch) diameter from the dried blood spot on the filter paper were used for DNA extraction, which was equivalent to 15–20 μL of whole blood. The punched-out filter paper circles were incubated in 30 μL of proteinase-K and 180 μL of incubation buffer from the kit at 70 °C for 90 minutes and then followed with the extraction instructions. The extracted DNA was eluted with 50 μL of elution buffer and preserved until use at −30 °C. PCR: For identification of *P*. *knowlesi*, partial *cytb* and partial *msp1* of *P*. *knowlesi* were amplified by nested PCR. For primary PCR of the *cytb*, real-time PCR was performed using a primer set of PCBF and PCBR. For secondary PCR of the *cytb*, conventional PCR was performed using a primer set of PKCBF and PKCBR. For primary PCR of the *msp1*, real-time PCR was performed using a primer set of Pk_MSP1_F3 and Pk_MSP1_R2. For secondary PCR of the *msp1*, conventional PCR was performed using a primer set of Pk_MSP1_F2 and Pk_MSP1_R2. For identification of *P*. *falciparum*, real-time PCR was performed using a primer set of Pf F1 and Pf R1. For identification of *P*. *vivax*, real-time PCR was performed using a primer set of PvF11 and PvR7. For identification of *P*. *ovale*, real-time PCR was performed using a primer set of POCBF and POCB_R1. For identification of *P*. *malariae*, real-time PCR was performed using a primer set of PMCBF and PMCBR. **Abbreviations:** *cytb*, cytochrome b gene; *msp1*, merozoite surface protein-1 gene.
(XLSX)
